# Initiation and duration of folic acid supplementation in preventing congenital malformations

**DOI:** 10.1186/s12916-023-03000-8

**Published:** 2023-08-07

**Authors:** Jing Dong, Lin-Liang Yin, Xue-Dong Deng, Chun-Ya Ji, Qi Pan, Zhong Yang, Ting Peng, Jiang-Nan Wu, for the Early Pregnancy Ultrasound Screening, Maternal Exposures and Congenital Malformation Risk collaborators, Gui-Hua Wu, Liu-Ying Zhou, Mei Li, Yue-Qin Chen, Jia-Xiang Yang, Bai-Song Liang, Tong Ru, Chun-Li Jing, Weng-Rong Zhou, Li Cao, Qin Li, Gui-Ping Li, Tai-Zhu Yang, Xin-Ru Gao, Li-Ling Shi, Yu-Qing Zhou, Xue-Qin Ji, Bo Liang, Qing Han, Ling Ren, Wen-Rong Wang, Guo-Wei Tao

**Affiliations:** 1 Medical Center of Diagnosis and Treatment for Cervical Disease, Obstetrics and Gynecology Hospital, Fudan University, Shanghai, 200011 China; 2 Center for Medical Ultrasound, The Affiliated Suzhou Hospital of Nanjing Medical University, Suzhou Municipal Hospital, Suzhou, 215006 China; 3 Department of Obstetrics, Obstetrics and Gynecology Hospital, Fudan University, Shanghai, 200011 China; 4 Department of Clinical Epidemiology, Obstetrics and Gynecology Hospital, Fudan University, Shanghai, 200011 China

**Keywords:** Folic acid supplementation, Congenital malformation, Heart defects, Neural tube defects, Cohort study

## Abstract

**Background:**

Folic acid (FA) supplementation is associated with a lower risk of the neural tube and heart defects and is recommended for women of childbearing age. Although there are detailed recommendations, differences in the initiation time and duration of FA supplementation remain poorly studied.

**Methods:**

A multicentre prospective study of 17,713 women was conducted. The incidence of congenital malformations in women taking a recommended dosage (e.g. 0.4 or 0.8 mg/day) of FA was compared with that in women without supplementation. The predicted probability of malformations by the initiation time and duration of FA use was estimated to determine optimal options.

**Results:**

Periconceptional FA supplementation was associated with a lower and insignificant risk of congenital malformations (1.59% vs. 2.37%; odds ratio [OR] 0.69; 95% confidence interval [CI]: 0.44–1.08), heart defects (3.8 vs. 8.0 per 1000 infants; OR, 0.47; 0.21–1.02), and neural tube defects (7.0 vs. 11.5 per 10,000 infants; OR, 0.64; 0.08–5.15). FA use after pregnancy provided greater protection against total malformations. Statistically significant associations were found in women who initiated FA supplementation in the first month of gestation (OR, 0.55; 95% CI: 0.33–0.91) and in those who supplemented for 1 to 2 months (OR, 0.59; 95% CI: 0.36–0.98). Similar results were found for heart defects. The optimal initiation time was 1.5 (optimal range: 1.1 to 1.9) months before pregnancy and a duration of 4.0 (3.7 to 4.4) months was reasonable to achieve the lowest risk of congenital malformations. Heart defect prevention required an earlier initiation (2.2 vs. 1.1 months before pregnancy) and a longer duration (4.7 vs. 3.7 months) than the prevention of other malformations.

**Conclusions:**

The timely initiation of FA supplementation for gestation was associated with a decreased risk of congenital malformations, which was mainly attributed to its protection against heart defects. The initiation of FA supplementation 1.5 months before conception with a duration of 4 months is the preferred option for congenital malformation prevention.

**Trial registration:**

Chictr.org.cn identifier: ChiCTR-SOC-17010976.

**Supplementary Information:**

The online version contains supplementary material available at 10.1186/s12916-023-03000-8.

## Background

Congenital malformations are structural or functional defects that occur during prenatal development and are associated with infantile short-term death and long-term disabilities [1]. It has been estimated that at least 7.9 million people are born with a birth defect every year worldwide and 3.3 million of them die annually before 5 years of age [2]. Although the exact mechanisms of the majority of congenital malformations remain unknown, some specific congenital anomalies could be prevented by periconceptional intervention, such as folic acid (FA) supplementation to protect against neural tube defects (NTDs) [3]. In addition to protection against NTDs, FA supplementation is associated with a lower risk of the cardiac, urinary tract, and limb defects [4–6]. Based on these primary prevention effects, periconceptional FA use has been recommended by the US government and the World Health Organization for all women of childbearing age since 1992 [7, 8].

The recommendations made FA widely available for women of childbearing age and have led to substantial achievements in preventing NTDs [3]. However, inconsistent and imprecise results were found for the difference in the initiation of therapy, and there are no studies on the duration of supplementation [3]. Furthermore, although the China Maternal and Child Health Association guidelines provide detailed recommendations on the initiation time and duration of FA supplementation [9], comparative studies of the preventive effects of FA supplementation in different scenarios are rarely reported.

Given that the sensitive and vulnerable early period of foetal development is between the 3^rd^ and the 8^th^ weeks of gestation [5] and that a sustained period (e.g. 12 weeks) of FA is required to achieve an effective red-blood-cell (RBC) folate level for attenuating the risk of NTDs [10, 11], the guideline recommended starting FA 3 months before conception or initiating supplementation immediately after pregnancy [9]. However, as most pregnant women in China have their first prenatal care visit after the 7^th^week of gestation [12], this may delay the initiation of FA. Inappropriate FA supplementation may reduce the effectiveness of prevention (e.g. inadequate supplementation) [13], increase the financial burden, or even endanger the health of the foetus (e.g. excessive supplementation), including increasing the risk of childhood asthma and congenital malformations [14, 15]. Therefore, the determination of the optimal initiation time and duration is critical for better protection efficacy and to prevent side effects from excessive supplementation [16]. We conducted a multicentre prospective cohort study to determine the optimal initiation time and duration of FA supplementation for preventing congenital malformations.

## Methods

### Study design

This was a multicentre prospective cohort study of early pregnancy ultrasound screening, maternal exposures and risk of congenital malformations. This investigation was conducted between August 2017 and August 2020 [17]. Pregnant women with a singleton pregnancy who underwent early pregnancy ultrasound examinations at 23 tertiary hospitals between 11 and 13 weeks of gestation were recruited from August 2017 to March 2019. Data on maternal general characteristics (e.g. age, education level, region, history of adverse pregnancy outcomes), periconceptional maternal exposure and early pregnancy complications and treatments (e.g. medicine exposure, vaginal bleeding, FA supplementation, or progesterone supplementation) were collected by trained clinicians through face-to-face interviews at recruitment.

Congenital malformations were diagnosed through routine prenatal and postnatal exams (e.g. ultrasound examinations, serum marker detections, physical examinations, and abnormality surveillance) and checked by reviewing medical records. We excluded women who were lost to follow-up from the analysis.

### Folic acid supplementation

During recruitment, women were surveyed regarding whether they received supplementation (e.g. FA alone or FA-containing multivitamin supplementation) by showing them a list of common FA products. Details (e.g. times and quantities of FA prescriptions) were extracted from the medical records to determine the initiation time and estimate the duration of supplementation. Daily intake of the recommended dosages (0.4 or 0.8 mg) of FA for at least 1 month was defined as having the supplementation. Since 12 weeks of supplementation is critical for effective folate concentrations and 3 months before pregnancy up to the third month of the pregnancy are key supplemental points in the guideline [9–11], we classified the women who took FA into three categories: preconception initiation for more than 3 months, preconception supplementation within 3 months, or FA supplementation after becoming pregnant according to the initiation time. The time of pregnancy was estimated based on a reliable last menstrual period and/or confirmed by the size of the gestational sac or embryo measured by ultrasound in early pregnancy. We also subgrouped the women by duration into groups with durations ≤ or > 3 months. Women without FA supplementation were referred to as the no supplement group.

No women in the no supplement group took periconceptional FA in the current pregnancy. Among the included women, 16 women whose supplementation information was not available were marked as the FA unknown group. These women and an additional 252 persons whose supplemental details were missing were excluded from the assessment of the association of the initiation time and duration of FA use with congenital malformations.

### Congenital malformations

Congenital malformations were diagnosed by a standardized protocol. Five routine exams were performed during the follow-up period, including serum testing for Down syndrome screening, first (11–13 weeks of gestation) and second trimester (24–28 weeks of gestation) ultrasound examinations, postpartum physical examinations (birth to discharge) and the birth defect monitoring (discharge to the sixth week after birth) [17, 18]. For those suspected of congenital malformations, further examinations, including detailed echocardiography, magnetic resonance imaging, serum/amniotic fluid genetic testing, or pathological anatomy, were performed to confirm the specifics of the malformations or to exclude a diagnosis. All malformations were adjudicated by a member of the quality control committee by reviewing medical records.

All malformations were coded according to the International Classification of Diseases, 10^th^ Revision. In the present study, cardiovascular anomalies (IDC-10 codes: Q20 to Q28) and NTD, including anencephaly (Q00.0 to Q00.1) and spina bifida (Q05.0 to Q05.9, Q07.01, and Q07.03), were considered secondary outcomes.

### Potential confounders

Data on variables that might be potential confounders were collected, including general characteristics (e.g. maternal age, region, ethnicity, maternal education level, family income level, conception mode, and parity), history of adverse pregnancy outcomes, early pregnancy conditions, and medicine exposure. A history of adverse pregnancy outcomes included miscarriage, ectopic pregnancy, infertility, induced labour, congenital malformations, or preterm birth in previous pregnancies. Early pregnant conditions referred to influenza/cold, a fever ≥ 38 ℃, skin rash, vaginal bleeding, thyroid dysfunction, gestational hyperglycaemia/diabetes, gastrointestinal diseases, and gynaecological inflammation or diseases.

### Data analyses

The incidence and 95% confidence intervals (95% CIs) were estimated for total and various organ malformations. Differences in the proportion of general characteristics and congenital malformations between women with and without FA supplementation were compared by chi-square or Fisher’s exact tests.

The primary analyses were conducted to study the association between FA category (e.g. yes vs. no; various FA groups vs. no FA) and the risk of congenital malformations. The analyses aimed to compare the difference in the effectiveness of FA supplementation in preventing congenital malformations at the main guideline-recommended initiation and duration of supplementation. Binary logistic regression was performed to estimate odds ratios (ORs) for congenital malformations in women with supplementation relative to those without supplementation. Adjusted ORs were evaluated after controlling for region, ethnicity, medicine exposure, maternal education level, family income level adverse pregnancy outcome history, early pregnancy conditions, conception mode, parity, and maternal age.

Sensitivity analyses restricted to natural conception, Han ethnicity, and women without a malformation history were performed to test the robustness of the results. Since a history of adverse pregnancy outcome (e.g. infertility [19], malformation history), gestational conditions (e.g. influenza) [20], medicine exposure [21], multiparity [22], and advanced maternal age [23] are associated with a higher risk of congenital malformations, stratification analyses were conducted according to these variables.

To compare the disparity in the protection of FA supplementation against congenital malformations between women with pre- and post-conception initiation, we evaluated the association according to the initiation of the supplementation (e.g. pre- or post-conception). Further analyses were performed in women who started FA before pregnancy (e.g. > or ≤ 3 months before pregnancy). In these analyses, we grouped the women according to the month they started FA supplementation to understand the change in the protective effect.

The secondary analyses focused on the association of continuous initiation and the duration of FA supplementation with congenital malformations. The malformation risks were compared among women with varying combinations of the initiation time and duration of FA supplementation. The association was further estimated through the nonlinear fitting of the predicted probability of binary logistic regression. The details of the fit curves were summarized, and the models were considered to be good if an *R*-squared value > 0.75 was obtained. The best options for the initiation time and duration were selected based on the lowest risk of malformations. Optional ranges in which the malformation risk was noninferior to the lowest level were identified, with a margin of 50% of the standard error.

The FA supplementation and reduction risk for heart defects and NTDs were estimated. Sensitivity and stratification analyses (pre- or post-conception FA) were conducted for heart defects but not for NTDs, as there was only one NTD in women without FA supplementation. There might be different optimal initiation times between heart defects and the other malformations. Therefore, further analyses on the FA initiation time and duration of FA supplementation were performed between heart defects and other malformations.

All analyses were conducted in SAS version 9.3, Microsoft Office Excel, and Stata (version 14.0, Stata Corp., College Station, TX, USA). A two-sided *P* < 0.05 was deemed statistically significant.

## Results

### General characteristics of the women

**Fig. 1:**
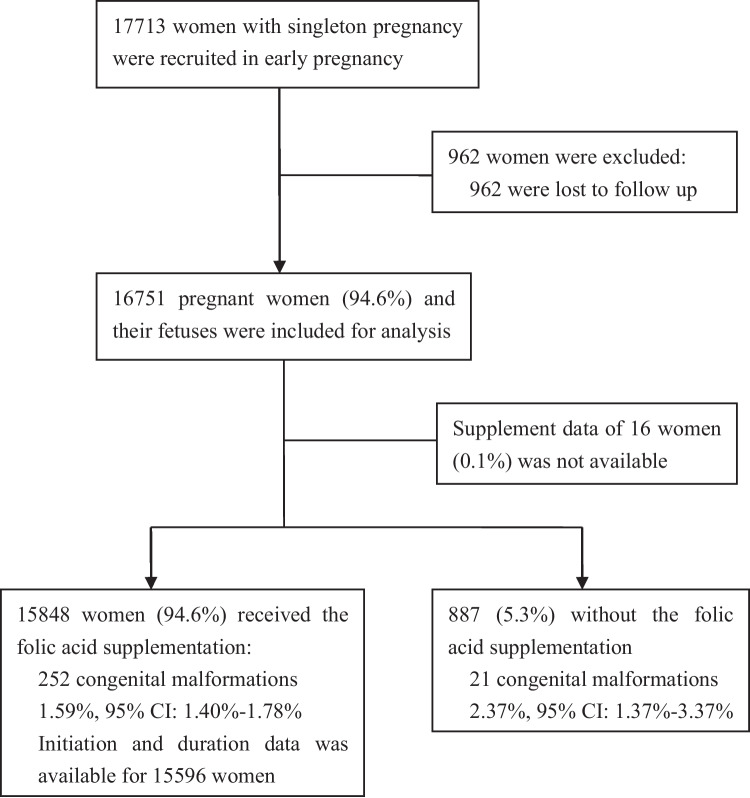
Flow chart of study

The main characteristics were balanced between the women who were included and excluded in the analysis (Additional file 1: Table S1). Among the 16,751 included women, 15,848 (94.6%) received FA (Fig. 1). The majority of the studied women was of Han ethnicity and had conceived naturally. Differences in all characteristics except for region and ethnicity were found between women with and without supplementation (Table 1).

**Table 1:** General characteristics and incidence of congenital malformations between women with and without folic acid supplementation

General characteristics and congenital malformations	Folic acid supplementation			
	Yes (*N* = 15,848)	No (*N* = 887)	Total (*N* = 16,735)	*P* value
Region				0.32
East	7900 (49.8)	442 (49.8)	8345	
West	4084 (25.8)	210 (23.7)	4301	
North	2974 (18.8)	176 (19.8)	3150	
Central	890 (5.6)	59 (6.7)	949	
Han ethnicity	15,119 (95.4)	845 (95.3)	15,964 (95.4)	0.67
Education level (years)				< 0.001
< 9	1426 (9.0)	137 (15.4)	1563 (9.3)	
10–12	2157 (13.6)	157 (17.7)	2314 (13.8)	
13–15	4044 (25.5)	241 (27.2)	4285 (25.6)	
≥ 16	8221 (51.9)	352 (39.7)	8573 (51.2)	
Family income (RMB Yuan per person per month)^a^				0.043
< 3000	537 (3.4)	35 (3.9)	572 (3.4)	
3000–5000	4936 (31.1)	308 (34.7)	5244 (31.3)	
5001–8000	5149 (32.5)	284 (32.0)	5433 (32.5)	
> 8000	4770 (30.1)	230 (25.9)	5000 (29.9)	
History of adverse pregnant outcomes^b^	2884 (18.2)	129 (14.5)	3013 (18.0)	0.006
Early-pregnant conditions^c^	6169 (38.9)	308 (34.7)	6477 (38.7)	0.012
Natural conception	14,649 (92.4)	862 (97.2)	15,511 (92.7)	< 0.001
Periconceptional medicine exposure	4221 (26.6)	194 (21.9)	4415 (26.4)	0.002
Nulliparous women	10,812 (68.2)	441 (49.7)	11,253 (67.2)	< 0.001
Maternal age (years)				0.001
< 25	1527 (9.6)	109 (12.3)	1636 (9.8)	
25–34	12,679 (80.0)	663 (74.7)	13,342 (79.7)	
≥ 35	1642 (10.4)	115 (13.0)	1757 (10.5)	
Congenital malformations				
Total, no. (%)	252 (1.6)	21 (2.4)	273 (1.6)	0.075
Heart defects, no. (per 1000 infants)	60 (3.8)	7 (7.9)	67 (4.0)	0.089
Neural tube defects, no. (per 10,000 infants)	11 (6.9)	1 (11.3)	12 (7.2)	0.47

^a^Information of family income was not available for 486 women

^b^Adverse pregnancy outcomes refer to abortion, ectopic pregnancy, infertility, induced labour, congenital malformations, or preterm birth in previous pregnancies

^c^Early pregnancy conditions mean influenza/cold, a fever ≥ 38℃, skin rash, vaginal bleeding, thyroid dysfunction, gestational hyperglycemia/diabetes, gastrointestinal diseases, gynaecological inflammation or diseases

At the end of follow-up, 273 congenital malformations were confirmed, with an incidence of 16.3 (95% CI, 14.5–18.3) per 1000 infants. Among them, 67 were heart defects (4.0 per 1000 infants, 95% CI: 3.2–5.1), and 12 NTDs were identified (7.0 per 10,000 infants, 95% CI: 3.0–11.0; Additional file 1: Table S2).

### FA supplementation and risk of congenital malformations

**Fig. 2:**
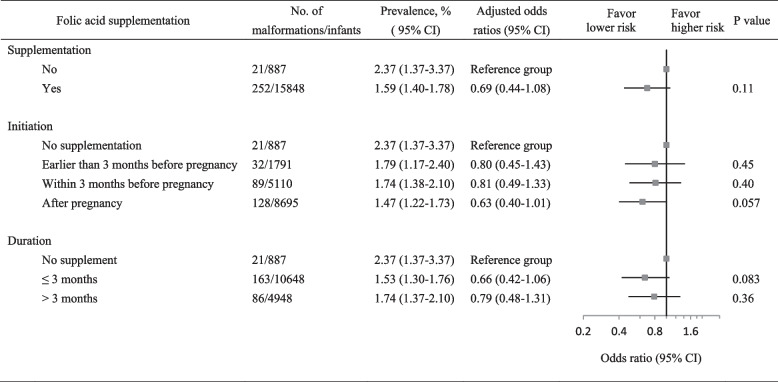
Association between folic acid supplementation and risk of congenital malformations. Odds ratios for the risk of malformations were estimated in women with folic acid compared with those without supplementation after controlling for region, maternal age, ethnicity, parity, conception mode, education level, family income, history of adverse pregnancy outcomes, medicine exposure, and early pregnancy conditions

Women with FA supplementation had a lower incidence of congenital malformations than those without the supplementation (1.59% vs. 2.37%; adjusted OR, 0.69; 95% CI: 0.44–1.08; Fig. 2). Compared with women without supplementation, women with FA initiated after becoming pregnant had the lowest risk (OR, 0.63; 95% CI: 0.40–1.01; Fig. 2). No significant differences in protection were found between women with FA supplementation duration of ≤ 3 and > 3 months (Fig. 2).

Sensitivity analyses that were restricted to women with Han ethnicity and those without a history of congenital malformations did not show significant changes. However, FA supplementation was associated with a decreased risk of congenital malformations in women who conceived naturally (OR, 0.63; 95% CI: 0.40–0.99; Additional file 1: Tables S3).

Stratification analyses indicated that no significant associations existed between variants of FA supplementation and congenital malformations in women with a history of adverse pregnancy outcomes, those who had a medicine exposure, multiparous women, and those with advanced age (e.g. > 35 years) (Additional file 1: Tables S4-S7). However, the protection effect of FA was detected in women with early pregnancy conditions (Additional file 1: Table S8).

### Pre- and post-conception FA supplementation

Compared with women without FA supplementation, the risk of congenital malformations was lower in women who started FA after pregnancy. The association was (marginally) statistically significant in the sensitive analyses (Additional file 1: Table S3). Further analyses indicated that, among women who initiated FA after becoming pregnant, an earlier initiation (e.g. the first month of gestation, OR, 0.55; 95% CI: 0.33–0.91) and a moderate duration (e.g. 1.01–2 months, OR, 0.59; 95% CI: 0.36–0.98) were associated with a reduced risk of congenital malformations relative to those without supplementation (Table 2). Preconceptional initiation of FA, including in subgroups of women who started the supplementation > or ≤ 3 months before pregnancy, was not significantly associated with congenital malformations (Additional file 1: Tables S9-11).

**Table 2:** Post-conception folic acid supplementation and risk of congenital malformations

Variants of folic acid supplementation	Risk of congenital malformations				
	No. of malformations	No. of infants	Prevalence,% (95% CI)	Adjusted odds ratio (95% CI)^a^	*P* value
Supplementation					
No	21	887	2.37 (1.37–3.37)	1.00	
Yes	128	8695	1.47 (1.22–1.73)	0.64 (0.40–1.03)	0.063
Initiation					
No supplement	21	887	2.37 (1.37–3.37)	1.00	
The first month	57	4430	1.29 (0.95–1.62)	0.55 (0.33–0.91)	0.02
The second month	51	3343	1.53 (1.11–1.94)	0.64 (0.38–1.07)	0.086
The third month	20	922	2.17 (1.23–3.11)	0.91 (0.49–1.70)	0.77
Duration					
No supplement	21	887	2.37 (1.37–3.37)	1.00	
1 month	33	1993	1.66 (1.10–2.22)	0.68 (0.39–1.19)	0.17
1.01 months ~	63	4572	1.38 (1.04–1.72)	0.59 (0.36–0.98)	0.041
2.01 months ~	32	2130	1.50 (0.99–2.02)	0.66 (0.38–1.17)	0.15

^a^Adjusted for region, maternal age, ethnicity, parity, conception mode, education level, family income, history of adverse pregnancy outcomes, medicine exposure, and early pregnancy conditions

### Optimal options for the initiation time and duration of FA supplementation

Among the various initiation time and duration combinations, women who started FA supplementation 2.5 months before pregnancy with a duration of 5.5 months, those who initiated FA 1.5 months before pregnancy with a duration of 4.5 months, and women who started FA after becoming pregnant and with a duration of 2.5 months had similar and better performance (Additional file 2: Figure S1).

**Fig. 3:**
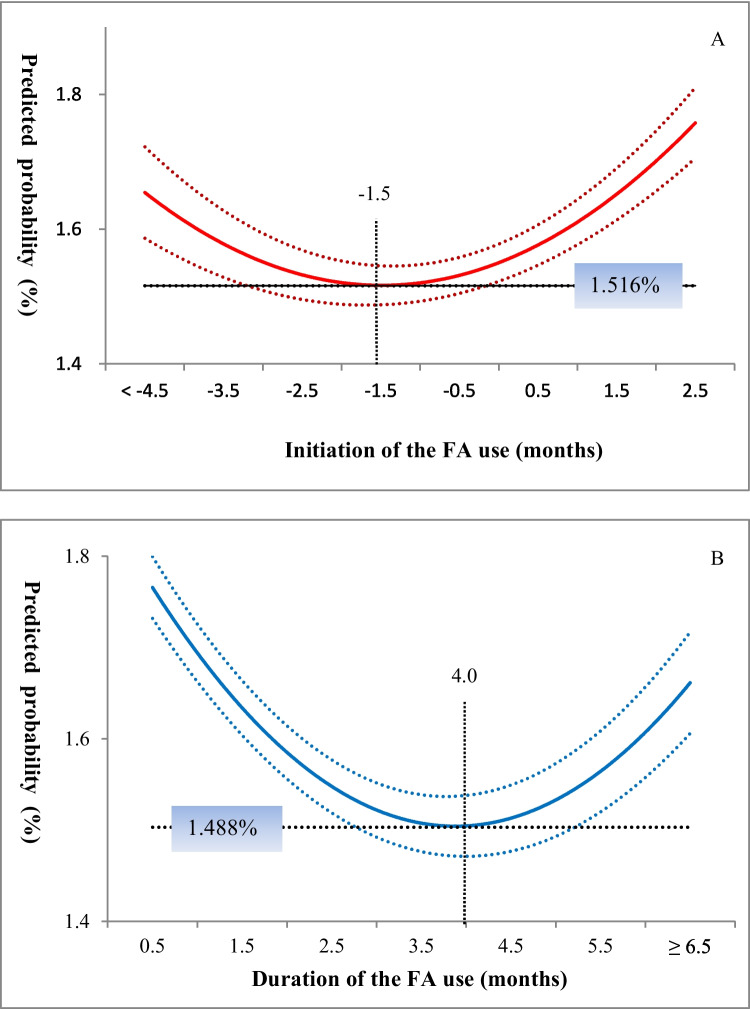
Predicted probability of congenital malformations by the initiation and duration of folic acid supplementation. The predicted probabilities of congenital malformations in women without folic acid supplementation were 2.35% and 2.37% in the initiation (**A**) and duration (**B**) analyses, respectively. The tolerable risk was 1.519% and 1.492% for the initiation and duration of folic acid supplementation, respectively

The initiation and duration of FA supplementation and the predicted probability of congenital malformations presented a well-fitted quadratic regression relationship (Additional file 1: Table S12). The best optimal initiation time of FA supplementation was 1.5 months before pregnancy, with an optional range from 1.1 to 1.9 months before pregnancy. During this period, women with FA supplementation had the lowest risk of congenital malformations at 1.52%. The optimal option for the duration of FA supplementation was 4.0 (3.7 to 4.4) months (Fig. 3).

### FA supplementation and risk of heart defects and NTDs

FA supplementation was marginally significantly associated with the risk of heart defects (3.8 vs. 8.0 per 1000 infants; adjusted OR, 0.47; 95% CI: 0.21–1.02; Table 3). Similar results were found in sensitivity analyses (Additional file 1: Tables S13). Women who initiated FA after pregnancy had a better preventive effect against heart defects than those who started FA supplementation before pregnancy (Table 4). FA supplementation started in the second month of gestation was significantly associated with a reduced risk of heart defects (OR, 0.37; 95% CI: 0.14–0.98). Although no significant associations were found, women who initiated FA between 2 and 1 month before pregnancy had the lowest risk of having an infant with a heart defect (OR, 0.29; 95% CI: 0.06–1.43; Table 4). FA supplementation was not associated with NTDs. However, women who started the supplementation after pregnancy had a 67% lower risk of NTDs relative to those without supplementation (Table 3).

**Table 3:** Folic acid supplementation and risk of heart defects and neural tube defects

Variants of folic acid supplementation	Risk of heart defects					Risk of neural tube defects				
	No. of malformations	No. of infants	Incidence, per 1000 infants (95% CI)	Adjusted odds ratio (95% CI)^a^	*P* value	No. of malformations	No. of infants	Incidence, per 10,000 infants (95% CI)	Adjusted odds ratio (95% CI)^a^	*P* value
Supplementation										
No	7	873	8.0 (2.1–13.9)	1.00		1	867	11.5 (1.1–34.2)	1.00	
Yes	60	15,656	3.8 (2.9–4.8)	0.47 (0.21–1.02)	0.056	11	15,607	7.0 (2.9–11.2)	0.64 (0.08–5.15)	0.68
Initiation										
No supplementation	7	873	8.0 (2.1–13.9)	1.00		1	867	11.5 (1.1–34.2)	1.00	
Earlier than 3 months before pregnancy	10	1769	5.7 (2.2–9.2)	0.80 (0.29–2.20)	0.66	1	1760	5.7 (1.0–16.8)	0.63 (0.03–11.69)	0.76
Within 3 months before pregnancy	18	5039	3.6 (1.9–5.2)	0.49 (0.20–1.21)	0.12	7	5028	13.9 (3.6–24.2)	1.62 (0.18–14.26)	0.66
After pregnancy	31	8598	3.6 (2.3–4.9)	0.45 (0.20–1.04)	0.061	3	8570	3.5 (1.0–12.0)	0.33 (0.03–3.22)	0.34
Duration										
No supplement	7	873	8.0 (2.1–13.9)	1.00		1	867	11.5 (1.1–34.2)	1.00	
≤ 3 months	41	10,526	3.9 (2.7–5.1)	0.49 (0.22–1.10)	0.084	6	10,491	5.7 (1.1–10.3)	0.55 (0.07–4.69)	0.59
> 3 months	18	4880	3.7 (2.0–5.4)	0.50 (0.20–1.24)	0.13	5	4867	10.0 (1.0–19.0)	1.05 (0.11–10.19)	0.97

^a^Adjusted for region, maternal age, ethnicity, parity, conception mode, education level, family income, history of adverse pregnancy outcomes, medicine exposure, and early pregnancy conditions

**Table 4:** Pre- and post-conception folic acid supplementation and risk of heart defects

Folic acid supplementation	Pre-conception FA use			Post-conception FA use		
	No. of malformations/infants	Adjusted odds ratio (95% CI)^a^	*P* value	No. of malformations/infants	Adjusted odds ratio (95% CI)^a^	*P* value
Supplementation						
No	7/873	1.00		7/873	1.00	
Yes	28/6808	0.68 (0.28–1.66)	0.40	31/8598	0.45 (0.20–1.03)	0.059
Initiation						
No supplementation	7/873	1.00		7/873	1.00	
Earlier than 4 months before pregnancy	3/559	0.99 (0.24–4.12)	0.99			
Between 4 and 3 months before pregnancy	7/1210	1.03 (0.34–3.14)	0.96		–	
Between 3 and 2 months before pregnancy	6/1466	0.67 (0.22–2.10)	0.49		–	
Between 2 and 1 month before pregnancy	2/1053	0.29 (0.06–1.43)	0.13		–	
Within 1 month before pregnancy	10/2520	0.60 (0.22–1.62)	0.31		–	
The first month of pregnancy		–		16/4389	0.45 (0.18–1.12)	0.085
The second month of pregnancy		–		10/3302	0.37 (0.14–0.98)	0.045
The third month of pregnancy		–		5/907	0.65 (0.20–2.06)	0.46
Duration						
No supplementation	7/873	1.00		7/873	1.00	
1 month		–		6/1966	0.36 (0.12–1.08)	0.68
1.01–2 months		–		15/4524	0.41 (0.17–1.03)	0.057
2.01–3 months	10/1928	0.79 (0.29–2.15)	0.64	10/2108	0.59 (0.22–1.59)	0.30
3.01–4 months	1/1200	0.12 (0.02–1.02)	0.052			
4.01–5 months	3/922	0.53 (0.13–2.11)	0.36			
5.01–6 months	9/1939	0.77 (0.27–2.19)	0.63			
> 6 months	5/819	1.18 (0.34–4.10)	0.79			

^a^Adjusted for region, maternal age, ethnicity, parity, conception mode, education level, family income, history of adverse pregnancy outcomes, medicine exposure, and early pregnancy conditions

Disparities in the optimal options for the initiation time and duration were found between heart defects and the other malformations: the options for initiation were 2.2 (1.7 to 2.7) and 1.1 (0.6 to 1.5) months before pregnancy, while the durations were 4.7 (4.2 to 5.1) and 3.7 (3.4 to 4.0) months, respectively (Additional file 2: Figures S2-S4). The models fit well, with all R-squared values > 0.80 (Additional file 1: Table S12).

## Discussion

### Principal findings of the study

Periconceptional FA supplementation had a protective effect against total congenital malformations and heart defects but not against NTDs. The reduction in the risk of congenital malformations reached significance when supplementation was initiated after pregnancy. The optimal initiation time of FA supplementation was 1.5 months before pregnancy with a 4-month duration to obtain the lowest incidence of malformations. An earlier initiation and a longer duration of FA supplementation were needed for heart defect prevention than for the prevention of other malformations.

### Context within previous literature

The association between FA supplementation and a reduced risk of NTDs was first reported by Smithells in 1980 [24]. Since then, this effect has been demonstrated by one randomized controlled trial and a substantial number of observational studies [25–29]. In the present study, women who initiated FA supplementation after pregnancy had a 67% lower risk of NTDs. However, FA supplementation was not associated with a significantly reduced risk of NTDs. This was consistent with the results of women in the southern region in previous studies in China [30, 31]. The process of neural tube closure is complex and is influenced by poor nutritional status, especially low folate intake. However, the prevention effect of FA supplementation is mainly significant in countries with the mandatory fortification of food with FA, and some NTDs are not preventable by FA supplementation [32].

In China, no mandatory folate fortification of food is performed. Dietary intake of folate may reduce the risk of NTDs and weaken the prevention effects of FA [33]. South China has a wider variety of foods and a better economic level than North China. Together with the disparity in dietary habits, pregnant women in South China are more likely to reach the effective threshold (25.5 nmol/L) for NTD prevention from their diet [34]. Previous studies have shown that the plasma folate levels in South China are higher than those in North (25.9 nmol/L vs. 13.3 nmol/L) [35]. Therefore, FA may be more effective in women in North China than in those in South China [31]. This may be the main reason for the null association found in the present study since only 18.8% of the subjects were from the northern region. In addition, as we recruited pregnant women mainly from tertiary care hospitals, the sample had a high proportion (76.8%) of women with an education level higher than high school. These women might also not have significant benefits from FA supplementation [36]. The sensitivity of different NTD types [31], and the small number of NTD cases due to inadequate sample size in the current study may also have contributed to the null association.

Although there are controversial correlations, the majority of evidence supports the conclusion that the FA use is associated with a decreased risk of heart defects [37]. In a recent study, the causal association between periconception RBC folate levels and heart desease in offspring was verified by the Mendelian randomization method [38]. In the Hungarian study, Czeizel contributed the unexpected reduction of birth defects to the preventative effect of FA supplementation against cardiovascular anomalies [5]. In this study, similar reduction risks of heart defects and total malformations were observed for some variants of FA supplementations. The mechanism remains poorly understood. Maternal FA supplementation may be involved in the intrauterine growth and development of the foetal heart through its effect on the methylation of genes associated with heart defects (e.g. the methylenetetrahydrofolate reductase (MTHFR), axis inhibitor 1 (AXIN1), and T-Box transcription factor 20 (TBX20) genes) [39].

In the current study, specific characteristics were found for the preventative effect of FA against heart defects. Compared with women who initiated FA after conception, the protection was similar for women who started FA within 3 months of conception. The optimal initiation time for the prevention of heart defects was 1 month earlier than that for the other malformations. This was consistent with the pooled result showing that long-term prepregnancy FA supplementation had the highest protection effect of heart defects [40].

Foetal heart development is sensitive to preconceptional medicine exposure, and higher target RBC folate levels are needed for heart defect prevention [38]. Serum plasma plateaued after 12 weeks of supplementation but RBC folate levels did not by the end of 24 weeks [41]. FA attenuates the increased heart defect risk induced by FA antagonists [42] and may reduce the teratogenic harm of high fever [43, 44]. In the current study, FA showed a protective effect against congenital malformations among women who had early pregnancy conditions but not for those with a healthy pregnancy. Therefore, an earlier initiation of FA supplementation may help to counteract the harms of prepregnancy exposure, protect against cardiovascular teratogenic effects and better achieve a higher and more effective RBC folate level. Further studies on reasons for this protection are warranted.

We identified that pregnant women who started FA supplementation 1.5 months before conception and had a 4-month duration had the best preventive effect against congenital malformations. This is in line with the results in a previous study [13]. In that study, compared with women who started FA 4–8 weeks after their last menstrual period, women who initiated FA supplementation 6 weeks before conception and who maintained it for 18 weeks (calculation from 4–8 weeks before pregnancy until the average gestation of 12.1 weeks at the first visit) had higher serum and RBC folate levels and a higher proportion of reaching an effective RBC folate level of > 906 nmol/L. Although the options for the initiation and duration varied, the optimal ending time of the supplementation was consistent, anchored at 10.7 to 11.1 weeks of gestation. This ending time of supplementation is convincing since the stage covers the most sensitive period of organogenesis in foetal development (e.g. between the 3^rd^ and 8^th^ week of gestation) [5, 45].

### Clinical implications of the findings

FA supplementation is a routine measure for the prevention of malformations in women of childbearing age in China [46, 47]. In contrast, the potential harms of FA are of high concern [3]. Excessive FA supplementation is associated with an increased risk of asthma and congenital malformations and might affect the long-term health outcomes of offspring [14, 15, 48]. Therefore, the initiation and duration of supplementation is critical for clinical practice in the context of cost–benefit balance and intervention safety. Our findings provide evidence for guiding the prescription of FA in obstetrics clinical practice and for improving the guidelines of FA supplementation. To better prevent congenital malformations, clinicians should evaluate the risk of malformations and promptly prescribe FA supplementation for women of childbearing age. Since prenatal care usually begins after the 7^th^ week of gestation, a feasible measure is to advise women to supplement FA for 1 month before starting to prepare for pregnancy.

An optimal duration of the FA supplementation is the key to achieve an effective level of RBC folate for the prevention of congenital malformations and to prevent the potential harms of excessive supplementations. We first identified the optimal option through quantitative analyses. This finding also presented economic significance for vulnerable women who are sensitive to the affordability of FA. Finally, the reasons for the disparity in the initiation time and duration between heart defects and the other malformations remain unknown. We could predict that the effective RBC folate level for the prevention of heart defects is approximately 1200 nmol/L, which is the level after 20 weeks of supplementation (equal to a duration of 4.7 months) of 400 µg [44].

### Strengths and limitations

Observation studies are the first choice in assessing the protective effect of FA [3]. In this multicentre prospective study, we accurately measured details of FA supplementation, prospectively and extensively collected data on congenital malformations, and assessed causality after controlling for many possible confounders. These merits ensure the accuracy and authenticity of the association. The large sample size provided statistical power to detect specific associations of variants of FA supplementation with the risk of congenital malformations.

There were some limitations in the present study. First, pregnant women were recruited at tertiary hospitals. The sample might have a better socialeconomic status [12]. These women might have better awareness and other factors that might be associated with the risk of malformations. In addition, the number of heart defects used to fit the initiation and duration of FA supplementation and the predicted probability was small and could therefore slightly affect the optimal values and their ranges. Thus, estimations of the effect of FA supplementation in preventing congenital malformations might be performed with caution and further studies with large sample sizes are warranted. Second, the 252 women whose details of FA supplementation were not available were considered to meet the definition and were included in the assessment of the association between FA and congenital malformations, which may have had an extremely slight effect on the results. Third, we were unable to further compare the preventive effect between the two doses (0.4 vs. 0.8 mg) since the data were not available. Finally, FA use was confirmed through medical records. The non-standardised medical records might lead to potential errors in the estimation of FA use among hospitals. In addition, although the regular intake rates were high (83.3 ~ 94.6%) in women who received FA [46, 49], we acknowledged that there might be different compliance of pill intake among the participants, thus inducing potential classification bias.

## Conclusions

The timely post-conceptional supplementation of 0.4 or 0.8 mg FA was associated with a lower risk of congenital malformations, mainly contributing to its protection against heart defects. Women who initiated FA supplementation 1.5 months before pregnancy and maintained it for 4 months had the best protective effect. An earlier initiation of FA supplementation is recommended for the prevention of heart defects. These findings provide evidence for guiding the prescription of FA in obstetrics clinical practice and for improving guidelines in balancing the cost–benefit and avoiding potential harms of excessive FA.

### Supplementary Information

**Additional file 1: Table S1. **General characteristics between included and excluded women. **Table S2.** Incidence of organ congenital malformations and its association with folic acid supplementation. **Table S3.** Sensitivity analyses for the association between folic acid and congenital malformations. **Table S4. **The association between folic acid supplement and congenital malformations according to history of adverse pregnancy outcomes. **Table S5.** The association between variants of folic acid supplementation and congenital malformations according to medicine exposure. **Table S6.** The association between folic acid supplementation and congenital malformations according to parity. **Table S7.** The association between folic acid supplementation and congenital malformations according to maternal age. **Table S8. **The association between folic acid supplementation and congenital malformations according to early pregnant conditions. **Table S9. **Pre-conception initiation of folic acid supplementation and congenital malformations. **Table S10. **Folic acid supplementation and risk of congenital malformations in women who started the supplementation earlier than three months before pregnancy. **Table S11.**  Folic acid supplementation and risk of congenital malformations in women who started the supplementation within three months before pregnancy. **Table S12. **Details of the fit curves between initiation and duration of folic acid supplementation and malformation risk. **Table S13. **Sensitivity analyses for the association between folic acid supplementation and risk of heart defects.

**Additional file 2: Figure S1. **Combinations of initiation and duration of folic acidsupplementation with the predicted probability of congenital malformations. **Figure S2.** Combinations of initiation and duration of folic acid with the predicted probability of heart defects and the other malformations. **Figure S3.** Predictive probability of heart defects and the other malformations by the initiation of folic acid supplementation. **Figure S4.** Predictive probability of heart defects and the other malformations by the duration of folic acid supplementation. 

## Data Availability

The data collected for the current study would be shared by contacting the corresponding author (JNW, wjnhmm@126.com) with reasonable request.

## Abbreviations

AXIN1: Axis inhibitor 1
CI: Confidence interval
FA: Folic acid
MTHFR: Methylenetetrahydrofolate reductase
NTD: Neural tube defect
OR: Odds ratio
RBC: Red blood cell
TBX20: T-Box transcription factor 20
