# Supplementary material for: Initiation and duration of folic acid supplementation in preventing congenital malformations
Source: BMC Med. 2023 Aug 7;21:292. doi: 10.1186/s12916-023-03000-8 (PMC10405478; doi:10.1186/s12916-023-03000-8)
Supplement: Supplementary file 1 — Additional file 1: Table S1. General characteristics between included and excluded women. Table S2. Incidence of organ congenital malformations and its association with folic acid supplementation. Table S3. Sensitivity analyses for the association between folic acid and congenital malformations. Table S4. The association between folic acid supplement and congenital malformations according to history of adverse pregnancy outcomes. Table S5. The association between variants of folic acid supplementation and congenital malformations according to medicine exposure. Table S6. The association between folic acid supplementation and congenital malformations according to parity. Table S7. The association between folic acid supplementation and congenital malformations according to maternal age. Table S8. The association between folic acid supplementation and congenital malformations according to early pregnant conditions. Table S9. Pre-conception initiation of folic acid supplementation and congenital malformations. Table S10. Folic acid supplementation and risk of congenital malformations in women who started the supplementation earlier than three months before pregnancy. Table S11. Folic acid supplementation and risk of congenital malformations in women who started the supplementation within three months before pregnancy. Table S12. Details of the fit curves between initiation and duration of folic acid supplementation and malformation risk. Table S13. Sensitivity analyses for the association between folic acid supplementation and risk of heart defects. [file 12916_2023_3000_MOESM1_ESM.docx]

**Initiation and duration of folic acid supplementation in preventing congenital malformations**

Dong J et al. BMC Med.

Additional fie 1

Table S 1. General characteristics between included and excluded women

| General characteristics | | Participants included for analysis | | |
| --- | --- | --- | --- | --- |
|  |  | No (%, N=962) | Yes (%, N=16751) | P value |
| Folic acid supplementation^*^ | |  |  | 0.56 |
|  | No | 50 (5.3) | 887 (5.3) |  |
|  | Yes | 910 (94.7) | 15848 (94.7) |  |
| Region^*^ | |  |  | <0.001 |
|  | Eastern | 343 (35.6) | 8345 (49.8) |  |
|  | Western | 196 (20.4) | 4301 (25.7) |  |
|  | Northern | 220 (22.9) | 3156 (18.8) |  |
|  | Central | 182 (18.9) | 949 (5.7) |  |
| Maternal age, years | |  |  | <0.001 |
|  | <25 | 142 (14.8) | 1637 (9.8) |  |
|  | 25-34 | 714 (74.2) | 13357 (79.7) |  |
|  | ≥ 35 | 106 (11.0) | 1757 (10.5) |  |
| Han ethnicity | | 911(94.7) | 15980 (95.4) | 0.32 |
| Maternal education level (years) | |  |  | 0.07 |
|  | ≤ 9 | 112 (11.6) | 1564 (9.3) |  |
|  | 10-12 | 139 (14.4) | 2318 (13.8) |  |
|  | 13-15 | 224 (23.3) | 4289 (25.6) |  |
|  | ≥ 16 | 487 (50.6) | 8580 (51.2) |  |
| Family income (RMB per person per month) ^*^ | |  |  | <0.001 |
|  | < 3000 | 42 (4.4) | 573 (3.4) |  |
|  | 3000-5000 | 353 (36.7) | 5248 (31.3) |  |
|  | 5001-8000 | 304 (31.6) | 5438 (32.5) |  |
|  | ≥8001 | 214 (22.2) | 5003 (29.9) |  |
| Parity=0 | | 616 (64.0) | 11266 (67.3) | 0.04 |
| Natural conception | | 931 (96.8) | 15527 (92.7) | <0.001 |
| History of adverse pregnant outcomes | | 157 (16.3) | 3016 (18.0) | 0.19 |
| Early-pregnancy conditions | | 356 (37.0) | 6479 (38.7) | 0.30 |
| Medicine exposure | | 238 (24.7) | 4417 (26.4) | 0.26 |

^*^ Data was not available of folic acid supplementation, region, and family income in 18, 21, and 538 women, respectively.

Table S 2. Incidence of organ congenital malformations and its association with folic acid supplementation

| Congenital malformations (ICD-10 codes) | No. of cases | Prevalence per 1000 infants (95% CI) | Folic acid supplementation | | Adjusted odds ratio (95%CI) |
| --- | --- | --- | --- | --- | --- |
|  |  |  | Yes | No |  |
|  |  |  | No. of cases/No. of infants | No. of cases/No. of infants |  |
| Nervous system (Q00 to Q07) | 31 | 1.9 (1.3-2.7) | 29/15625 | 2/868 | 0.80 (0.19-3.42) |
| Eye, ear, face, and neck (Q10 to 18) | 9 | 0.5 (0.3-1.0) | 8/15604 | 1/867 | 0.35 (0.04-2.92) |
| Circulatory system (Q20 to Q28) | 67 | 4.0 (3.2-5.1) | 60/15656 | 7/873 | 0.47 (0.21-1.02) |
| Respiratory and digstive system (Q30 to Q45) | 21 | 1.3 (0.9-2.0) | 18/15614 | 0/866 | -- |
| Genital organs (Q50 to Q56) and urinary system (Q60 to Q64) | 24 | 1.4 (0.9-2.1) | 27/15623 | 0/866 | -- |
| Musculoskeletal system (Q65 to Q79) | 38 | 2.3 (1.7-3.1) | 36/15632 | 2/868 | 0.97 (0.23-4.10) |
| Integumentary and other (Q80 to Q84) | 15 | 0.9 (0.5-1.5) | 13/15609 | 2/868 | 0.38 (0.08-1.73) |
| Other (Q85 to Q99) | 46 | 2.7 (2.0-3.6) | 41/15637 | 5/871 | 0.47 (0.18-1.22) |
| Neural tube defects (Q00 and Q05) | 12 | 0.7 (0.3-1.1) | 11/15607 | 1/867 | 0.64 (0.08-5.15) |
| Unknown | 40 | 2.4 (1.8-3.3) | 36/15632 | 4/870 | 0.57 (0.20-1.65) |

^*^ Adjusted for region, maternal age, ethnicity, parity, conception mode, education level, family income, history of adverse pregnancy outcomes, medicine exposure, and early pregnancy conditions.

Table S3. Sensitivity analyses for the association between folic acid and congenital malformations

| Variants of folic acid supplementation | Adjusted odds ratio (95% CI) | | | | | | | | |
| --- | --- | --- | --- | --- | --- | --- | --- | --- | --- |
|  | No. of malformations/infants | Women with natural conception | P value | No. of malformations/infants | Han ethnicity | P value | No. of malformations/infants | Women without a congenital anomalies history | P value |
| Supplementation |  |  |  |  |  |  |  |  |  |
| No | 21/862 | 1.00 |  | 20/845 | 1.00 |  | 21/875 | 1.00 |  |
| Yes | 229/14649 | 0.63 (0.40-0.99) | 0.043 | 242/15119 | 0.70 (0.44-1.11) | 0.13 | 249/15578 | 0.68 (0.43-1.07) | 0.097 |
| Initiation |  |  |  |  |  |  |  |  |  |
| No supplementation | 21/862 | 1.00 |  | 20/845 | 1.00 |  | 21/875 | 1.00 |  |
| Earlier than 3 months before pregnancy | 26/1409 | 0.87 (0.48-1.58) | 0.64 | 29/1727 | 0.74 (0.40-1.34) | 0.31 | 32/1710 | 0.83 (0.46-1.48) | 0.52 |
| Within 3 months before pregnancy | 78/4574 | 0.80 (0.48-1.31) | 0.37 | 88/4868 | 0.83 (0.50-1.37) | 0.47 | 87/5013 | 0.80 (0.49-1.30) | 0.36 |
| After pregnancy | 122/8420 | 0.61 (0.38-0.98) | 0.04 | 122/8283 | 0.63 (0.39-1.02) | 0.06 | 127/8606 | 0.63 (0.39-1.00) | 0.05 |
| Duration |  |  |  |  |  |  |  |  |  |
| No supplementation | 21/862 | 1.00 |  | 20/845 | 1.00 |  | 21/875 | 1.00 |  |
| 0.1~3 months | 155/10229 | 0.64 (0.40-1.03) | 0.06 | 156/10138 | 0.66 (0.41-1.07) | 0.09 | 161/10530 | 0.65 (0.41-1.04) | 0.07 |
| > 3 months~ | 71/4174 | 0.79 (0.48-1.31) | 0.37 | 83/4740 | 0.79 (0.47-1.31) | 0.35 | 85/4799 | 0.80 (0.48-1.31) | 0.37 |

All potential factors, including region, maternal age, ethnicity, parity, conception mode, education level, family income, history of adverse pregnancy outcomes, medicine exposure, and early pregnancy conditions, were included in the adjusted models except for the restricting factor.

Table S4. The association between folic acid supplement and congenital malformations according to history of adverse pregnancy outcomes.

| Variants of folic acid supplement | | Had an adverse outcome history | | | No adverse outcomes | | |
| --- | --- | --- | --- | --- | --- | --- | --- |
|  |  | No. of malformations/infants | Adjusted odds ratio (95% CI) ^a^ | P value | No. of malformations/infants | Adjusted odds ratio (95% CI) ^a^ | P value |
| History of supplement | |  |  |  |  |  |  |
|  | No | 2/129 | 1 |  | 19/758 | 1 |  |
|  | Yes | 46/2884 | 1.17 (0.28-4.98) | 0.83 | 206/12964 | 0.66 (0.41-1.06) | 0.087 |
| Initiation time | |  |  |  |  |  |  |
|  | No supplement | 2/129 | 1 |  | 19/758 | 1 |  |
|  | Earlier than 3 months before pregnancy | 10/560 | 1.09 (0.23-5.20) | 0.92 | 22/1231 | 0.75 (0.40-1.42) | 0.38 |
|  | Within 3 months before pregnancy | 18/1063 | 1.09 (0.25-4.84) | 0.91 | 71/4047 | 0.75 (0.44-1.26) | 0.22 |
|  | After pregnancy | 18/1220 | 0.98 (0.22-4.30) | 0.98 | 110/7475 | 0.58 (0.36-0.96) | 0.033 |
| Duration | |  |  |  |  |  |  |
|  | No supplement | 2/129 | 1 |  | 19/758 | 1 |  |
|  | ≤ 3 months | 24/1591 | 1.00 (0.23-4.30) | 0.99 | 139/9057 | 0.61 (0.38-1.00) | 0.05 |
|  | > 3 months | 22/1252 | 1.10 (0.25-4.86) | 0.9 | 64/3696 | 0.73 (0.43-1.23) | 0.24 |

Adjusted for region, maternal age, ethnicity, parity, conception mode, education level, family income, medicine exposure, and early pregnancy conditions.

Table S5. The association between variants of folic acid supplementation and congenital malformations according to medicine exposure.

| Variants of folic acid supplement | | Medicine exposure | | | No medicine exposure | | |
| --- | --- | --- | --- | --- | --- | --- | --- |
|  |  | No. of malformations/infants | Adjusted odds ratio (95% CI) ^a^ | P value | No. of malformations/infants | Adjusted odds ratio (95% CI) ^a^ | P value |
| History of supplement | |  |  |  |  |  |  |
|  | No | 4/194 | 1.00 |  | 17/693 | 1.00 |  |
|  | Yes | 89/4221 | 1.03 (0.37-2.86) | 0.96 | 163/11627 | 0.62 (0.37-1.04) | 0.071 |
| Initiation time | |  |  |  |  |  |  |
|  | No supplement | 4/194 | 1.00 |  | 17/693 | 1.00 |  |
|  | Earlier than 3 months before pregnancy | 10/519 | 0.92 (0.28-3.03) | 0.89 | 22/1272 | 0.78 (0.40-1.51) | 0.46 |
|  | Within 3 months before pregnancy | 35/1376 | 1.25 (0.44-3.59) | 0.67 | 54/3734 | 0.66 (0.37-1.15) | 0.14 |
|  | After pregnancy | 43/2272 | 0.91 (0.32-2.58) | 0.87 | 85/6423 | 0.56 (0.33-0.94) | 0.03 |
| Duration | |  |  |  |  |  |  |
|  | No supplement | 4/194 | 1.00 |  | 17/693 | 1.00 |  |
|  | ≤ 3 months | 58/2764 | 1.02 (0.36-2.83) | 0.98 | 105/7884 | 0.57 (0.34-0.95) | 0.033 |
|  | > 3 months | 30/1403 | 1.02 (0.35-2.96) | 0.97 | 56/3545 | 0.71 (0.41-1.25) | 0.24 |

Adjusted for region, maternal age, ethnicity, parity, conception mode, education level, family income, history of adverse pregnancy outcomes, and early pregnancy conditions.

Table S 6. The association between folic acid supplementation and congenital malformations according to parity.

| Variants of folic acid supplement | | Parity=0 | | | Parity≥ 1 | | |
| --- | --- | --- | --- | --- | --- | --- | --- |
|  |  | No. of malformations/infants | Adjusted odds ratio (95% CI) ^a^ | P value | No. of malformations/infants | Adjusted odds ratio (95% CI) ^a^ | P value |
| History of supplement | |  |  |  |  |  |  |
|  | No | 11/441 | 1.00 |  | 10/446 | 1.00 |  |
|  | Yes | 154/10812 | 0.55 (0.30-1.03) | 0.061 | 98/5036 | 0.85 (0.44-1.66) | 0.64 |
| Initiation time | |  |  |  |  |  |  |
|  | No supplement | 11/441 | 1.00 |  | 10/446 | 1.00 |  |
|  | Earlier than 3 months before pregnancy | 24/1488 | 0.64 (0.31-1.35) | 0.24 | 8/303 | 0.99 (0.38-2.59) | 0.98 |
|  | Within 3 months before pregnancy | 61/3896 | 0.62 (0.32-1.20) | 0.16 | 28/1214 | 0.98 (0.47-2.06) | 0.96 |
|  | After pregnancy | 67/5259 | 0.49 (0.26-0.94) | 0.03 | 61/3436 | 0.76 (0.39-1.51) | 0.44 |
| Duration | |  |  |  |  |  |  |
|  | No supplement | 11/441 | 1.00 |  | 10/446 | 1.00 |  |
|  | ≤ 3 months | 84/6653 | 0.49 (0.26-0.93) | 0.029 | 79/3995 | 0.85 (0.44-1.66) | 0.64 |
|  | > 3 months | 68/3990 | 0.68 (0.35-1.31) | 0.25 | 18/958 | 0.72 (0.33-1.61) | 0.43 |

Adjusted for region, maternal age, ethnicity, conception mode, education level, family income, history of adverse pregnancy outcomes, medicine exposure, and early pregnancy conditions.

Table S 7. The association between folic acid supplementation and congenital malformations according to maternal age.

| Variants of folic acid supplementation | | < 25 years old | | | 25-34 years old | | | ≥ 35 years old | | |
| --- | --- | --- | --- | --- | --- | --- | --- | --- | --- | --- |
|  |  |  | Adjusted odds ratio (95% CI) | P value |  | Adjusted odds ratio (95% CI) | P value |  | Adjusted odds ratio (95% CI) | P value |
| Supplementation | |  |  |  |  |  |  |  |  |  |
|  | No | 3/109 | 1 |  | 14/663 | 1 |  | 4/115 | 1 |  |
|  | Yes | 20/1527 | 0.46 (0.13-1.64) | 0.23 | 186/12679 | 0.67 (0.39-1.17) | 0.16 | 46/1642 | 0.94 (0.32-2.75) | 0.92 |
| Initiation | |  |  |  |  |  |  |  |  |  |
|  | No supplement | 3/109 | 1 |  | 14/663 | 1 |  | 4/115 | 1 |  |
|  | Earlier than 3 months before pregnancy | 1/73 | 0.59 (0.06-5.92) | 0.65 | 24/1478 | 0.78 (0.39-1.54) | 0.47 | 7/240 | 1.13 (0.30-4.17) | 0.86 |
|  | 3 months before pregnancy | 4/354 | 0.42 (0.09-1.94) | 0.27 | 71/4296 | 0.79 (0.44-1.43) | 0.44 | 14/460 | 1.06 (0.33-3.42) | 0.91 |
|  | Since pregnancy | 14/1067 | 0.43 (0.12-1.57) | 0.2 | 89/6704 | 0.61 (0.35-1.08) | 0.09 | 25/924 | 0.82 (0.28-2.43) | 0.72 |
| Duration | |  |  |  |  |  |  |  |  |  |
|  | No supplement | 3/109 | 1 |  | 14/663 | 1 |  | 4/115 | 1 |  |
|  | 0.1~3 month | 16/1258 | 0.43 (0.12-1.53) | 0.19 | 116/8285 | 0.65 (0.37-1.14) | 0.13 | 31/1105 | 0.87 (0.30-2.54) | 0.8 |
|  | > 3 months | 3/236 | 0.48 (0.09-2.50) | 0.38 | 68/4193 | 0.76 (0.42-1.39) | 0.38 | 15/519 | 1.03 (0.32-3.32) | 0.97 |

Adjusted for region, ethnicity, parity, conception mode, education level, family income, history of adverse pregnancy outcomes, medicine exposure, and early pregnancy conditions.

Table S 8. The association between folic acid supplementation and congenital malformations according to early pregnant conditions.

| Variants of folic acid supplement | | Had early pregnant conditions | | | No early pregnant conditions | | |
| --- | --- | --- | --- | --- | --- | --- | --- |
|  |  | No. of malformations/infants | Adjusted odds ratio (95% CI) ^a^ | P value | No. of malformations/infants | Adjusted odds ratio (95% CI) ^a^ | P value |
| History of supplement | |  |  |  |  |  |  |
|  | No | 11/308 | 1.00 |  | 10/579 | 1.00 |  |
|  | Yes | 119/6169 | 0.54 (0.29-1.03) | 0.062 | 133/9679 | 0.86 (0.45-1.65) | 0.65 |
| Initiation time | |  |  |  |  |  |  |
|  | No supplement | 11/308 | 1.00 |  | 10/579 | 1.00 |  |
|  | Earlier than 3 months before pregnancy | 14/690 | 0.56 (0.24-1.27) | 0.16 | 18/1101 | 1.01 (0.46-2.25) | 0.98 |
|  | Within 3 months before pregnancy | 49/1929 | 0.73 (0.37-1.44) | 0.36 | 40/3181 | 0.79 (0.39-1.59) | 0.50 |
|  | After pregnancy | 55/3470 | 0.44 (0.23-0.85) | 0.015 | 73/5225 | 0.83 (0.42-1.61) | 0.57 |
| Duration | |  |  |  |  |  |  |
|  | No supplement | 11/307 | 1.00 |  | 10/579 | 1.00 |  |
|  | ≤ 3 months | 76/4178 | 0.51 (0.26-0.97) | 0.039 | 87/6470 | 0.80 (0.41-1.55) | 0.51 |
|  | > 3 months | 42/1911 | 0.60 (0.30-1.20) | 0.15 | 44/3037 | 0.91 (0.45-1.85) | 0.80 |

Adjusted for region, maternal age, ethnicity, parity, conception mode, education level, family income, history of adverse pregnancy outcomes, and medicine exposure.

Table S9 Pre-conception initiation of folic acid supplementation and congenital malformations

| Folic acid containing multivitamin supplementation | | Risk of congenital malformations | | | | |
| --- | --- | --- | --- | --- | --- | --- |
|  |  | No. of cases | No. of infants | Prevalence, % (95% CI) | Adjusted odds ratio (95% CI) ^*^ | P value |
| Supplementation | |  |  |  |  |  |
|  | No | 21 | 887 | 2.37 (1.37-3.37) | 1.00 |  |
|  | Yes | 121 | 6901 | 1.75 (1.44-2.06) | 0.80 (0.49-1.30) | 0.36 |
| Initiation | |  |  |  |  |  |
|  | No supplementation | 21 | 887 | 2.37 (1.37-3.37) | 1.00 |  |
|  | Earlier than 3 months before pregnancy | 32 | 1791 | 1.79 (1.17-2.40) | 0.88 (0.49-1.60) | 0.67 |
|  | Within 3 months before pregnancy | 89 | 5110 | 1.74 (1.38-2.10) | 0.85 (0.51-1.41) | 0.53 |
| Duration | |  |  |  |  |  |
|  | No supplementation | 21 | 887 | 2.37 (1.37-3.37) | 1.00 |  |
|  | ≤ 3 months | 35 | 1953 | 1.79 (1.2-2.38) | 0.87 (0.49-1.53) | 0.63 |
|  | > 3 months | 86 | 4948 | 1.74 (1.37-2.10) | 0.85 (0.51-1.42) | 0.54 |

^*^ Adjusted for region, maternal age, ethnicity, parity, conception mode, education level, family income, history of adverse pregnancy outcomes, medicine exposure, and early pregnancy conditions.

Table S 10. Folic acid supplementation and risk of congenital malformations in women who started the supplementation earlier than three months before pregnancy

| Folic acid containing multivitamin supplementation | | Risk of congenital malformations | | | | |
| --- | --- | --- | --- | --- | --- | --- |
|  |  | No. of cases | No. of infants | Prevalence, % (95% CI) | Adjusted odds ratio (95% CI) ^*^ | P value |
| Supplementation | |  |  |  |  |  |
|  | No | 21 | 887 | 2.37 (1.37-3.37) | 1.00 |  |
|  | Yes | 32 | 1791 | 1.79 (1.17-2.40) | 0.99 (0.52-1.88) | 0.97 |
| Initiation | |  |  |  |  |  |
|  | No supplementation | 21 | 887 | 2.37 (1.37-3.37) | 1.00 |  |
|  | Earlier than 4 months before pregnancy | 7 | 563 | 1.24 (0.33-2.16) | 0.68 (0.27-1.74) | 0.42 |
|  | Between 4 and 3 months before pregnancy | 25 | 1228 | 2.04 (1.24-2.83) | 1.11 (0.57-2.17) | 0.76 |
| Duration | |  |  |  |  |  |
|  | No supplementation | 21 | 887 | 2.37 (1.37-3.37) | 1.00 |  |
|  | 5.01-6 months | 21 | 966 | 2.17 (1.25-3.10) | 1.17 (0.59-2.31) | 0.66 |
|  | > 6 months | 11 | 825 | 1.33 (0.55-2.12) | 0.72 (0.31-1.65) | 0.44 |

^*^ Adjusted for region, maternal age, ethnicity, parity, conception mode, education level, family income, history of adverse pregnancy outcomes, medicine exposure, and early pregnancy conditions.

Table S11. Folic acid supplementation and risk of congenital malformations in women who started the supplementation within three months before pregnancy

| Folic acid containing multivitamin supplementation | | Risk of congenital malformations | | | | |
| --- | --- | --- | --- | --- | --- | --- |
|  |  | No. of cases | No. of infants | Prevalence, % (95% CI) | Adjusted odds ratio (95% CI) ^*^ | P value |
| Supplementation | |  |  |  |  |  |
|  | No | 21 | 887 | 2.37 (1.37-3.37) | 1 |  |
|  | Yes | 89 | 5110 | 1.74 (1.38-2.10) | 0.82 (0.49-1.36) | 0.43 |
| Initiation | |  |  |  |  |  |
|  | No supplementation | 21 | 887 | 2.37 (1.37-3.37) | 1 |  |
|  | Between 3 and 2 months before pregnancy | 31 | 1491 | 2.08 (1.35-2.80) | 0.99 (0.55-1.79) | 0.97 |
|  | Between 2 and 1 month before pregnancy | 13 | 1064 | 1.22 (0.56-1.88) | 0.56 (0.28-1.16) | 0.12 |
|  | Within 1 month before pregnancy | 45 | 2555 | 1.76 (1.25-2.27) | 0.83 (0.48-1.43) | 0.51 |
| Duration | |  |  |  |  |  |
|  | No supplementation | 21 | 887 | 2.37 (1.37-3.37) | 1 |  |
|  | 2.01-3 months | 35 | 1953 | 1.79 (1.20-2.38) | 0.86 (0.49-1.52) | 0.60 |
|  | 3.01-4 months | 18 | 1217 | 1.48 (0.80-2.16) | 0.66 (0.34-1.28) | 0.22 |
|  | 4.01-5 months | 19 | 938 | 2.03 (1.12-2.93) | 0.97 (0.51-1.88) | 0.94 |
|  | 5.01-6 months | 17 | 1002 | 1.70 (0.90-2.50) | 0.78 (0.39-1.54) | 0.47 |

^*^ Adjusted for region, maternal age, ethnicity, parity, conception mode, education level, family income, history of adverse pregnancy outcomes, medicine exposure, and early pregnancy conditions.

Table S12. Details of the fit curves between initiation and duration of folic acid supplementation and malformation risk

| Type of exposure | Outcome | Equations | R-square | Optimal option | Lowest risk level | Corresponding figures |
| --- | --- | --- | --- | --- | --- | --- |
| Initiation | Total malformations | y=0.015(x+5.5)^2^-0.121(x+5.5)+1.760 | 0.916 | -1.5 | 1.52% | Figure 3 A |
| Duration | Total malformations | y=0.022(x+0.5)^2^-0.2(x+0.5)+1.943 | 0.904 | 4 | 1.49% | Figure 3 B |
| Initiation | Heart defects | y=0.033(x+5.5)^2^-0.219(x+5.5)+3.987 | 0.936 | -2.2 | 3.624 per 1000 | Figure S3 A |
| Duration | Heart defects | y=0.047(x+0.5)^2^-0.485(x+0.5)+4.816 | 0.846 | 4.7 | 3.565 per 1000 | Figure S4 A |
| Initiation | The other malformations | y=0.011(x+5.5)^2^-0.096(x+5.5)+1.372 | 0.919 | -1.1 | 1.16% | Figure S3 B |
| Duration | The other malformations | y=0.018(x+0.5)^2^-0.152(x+0.5)+1.469 | 0.908 | 3.7 | 1.15% | Figure S4 B |

Table S13. Sensitivity analyses for the association between folic acid supplementation and risk of heart defects

| Variants of folic acid supplementation | Adjusted odds ratio (95% CI) | | | | | | | | |
| --- | --- | --- | --- | --- | --- | --- | --- | --- | --- |
|  | No. of malformations/infants | Women with natural conception | P value | No. of malformations/infants | Han ethnicity | P value | No. of malformations/infants | Women without a congenital anomalies history | P value |
| Supplementation |  |  |  |  |  |  |  |  |  |
| No | 7/848 | 1.00 |  | 6/831 | 1.00 |  | 7/861 | 1.00 |  |
| Yes | 56/14476 | 0.49 (0.22-1.09) | 0.079 | 58/14935 | 0.55 (0.23-1.29) | 0.17 | 59/15388 | 0.48 (0.22-1.06) | 0.069 |
| Initiation |  |  |  |  |  |  |  |  |  |
| No supplementation | 7/848 | 1.00 |  | 6/831 | 1.00 |  | 7/861 | 1.00 |  |
| Earlier than 3 months before pregnancy | 9/1392 | 0.91 (0.33-2.56) | 0.86 | 9/1707 | 0.80 (0.27-2.36) | 0.69 | 10/1688 | 0.82 (0.30-2.28) | 0.71 |
| Within 3 months before pregnancy | 17/4513 | 0.52 (0.21-1.28) | 0.15 | 18/4798 | 0.56 (0.22-1.46) | 0.24 | 17/4943 | 0.47 (0.19-1.15) | 0.09 |
| After pregnancy | 29/8327 | 0.43 (0.19-0.99) | 0.048 | 30/8191 | 0.51 (0.21-1.24) | 0.14 | 31/8510 | 0.45 (0.20-1.03) | 0.059 |
| Duration |  |  |  |  |  |  |  |  |  |
| No supplementation | 7/848 | 1.00 |  | 6/831 | 1.00 |  | 7/861 | 1.00 |  |
| 0.1~3 months | 38/10112 | 0.47 (0.21-1.06) | 0.067 | 40/10022 | 0.55 (0.23-1.32) | 0.18 | 41/10410 | 0.49 (0.22-1.09) | 0.08 |
| > 3 months~ | 17/4120 | 0.56 (0.23-1.40) | 0.22 | 17/4674 | 0.53 (0.20-1.40) | 0.20 | 17/4731 | 0.47 (0.19-1.19) | 0.11 |

All potential factors, including region, maternal age, ethnicity, parity, conception mode, education level, family income, history of adverse pregnancy outcomes, medicine exposure, and early pregnancy conditions, were included in the adjusted models except for the restricting factor.
