# Supplementary material for: Initiation and duration of folic acid supplementation in preventing congenital malformations
Source: BMC Med. 2023 Aug 7;21:292. doi: 10.1186/s12916-023-03000-8 (PMC10405478; doi:10.1186/s12916-023-03000-8)
Supplement: Supplementary file 2 — Additional file 2: Figure S1. Combinations of initiation and duration of folic acidsupplementation with the predicted probability of congenital malformations. Figure S2. Combinations of initiation and duration of folic acid with the predicted probability of heart defects and the other malformations. Figure S3. Predictive probability of heart defects and the other malformations by the initiation of folic acid supplementation. Figure S4. Predictive probability of heart defects and the other malformations by the duration of folic acid supplementation. [file 12916_2023_3000_MOESM2_ESM.docx]

**Initiation and duration of folic acid supplementation in preventing congenital malformations**

Dong J et al. BMC Med.

Additional fie 2

Figure S1. Combinations of initiation and duration of folic acid supplementation with the predicted probability of congenital malformations

The circle represents the mean of the predicted probability of congenital malformations and the error bar represents the 95% confidence interval.

The red dotted line indicates the upper limit of the 95% CI in women who initiated folic acid after pregnancy and maintained 2.5 months of duration.

Figure S2. Combinations of initiation and duration of folic acid with the predicted probability of heart defects and the other malformations

Figure S2 A. Heart defects; Figure S2 B. the other malformations.

The circle represents the mean of the predicted probability of congenital malformations and the error bar represents the 95% confidence interval.

The red dotted line indicates the upper limit of the 95% CI in women who initiated folic acid after pregnancy and maintained 2.5 months of duration.

-2.2

Figure S3. Predictive probability of heart defects and the other malformations by the initiation of folic acid supplementation

The predicted probability of heart defects was 7.96 per 1000 infants and 1.59% for the other malformations in women without the FAS, respectively.

Figure S3 A congenital heart defects; Figure S3 B malformations other than heart defects.

The tolerable risk was 3.634 per 1000 infants and 1.165% for the initiation and duration of folic acid supplementation, respectively.

Controlling for region, maternal age, ethnicity, parity, conception mode, education level, family income, history of adverse pregnancy outcomes, medicine exposure, and early pregnancy conditions..

4.7

B

Figure S4. Predictive probability of heart defects and the other malformations by the duration of folic acid supplementation

The predicted probability of heart defects was 7.96 per 1000 infants and 1.59% for the other malformations in women without the FAS, respectively.

Figure S4 A heart defects; Figure S4 B the other malformations

The tolerable risk was 3.575 per 1000 infants and 1.151% for the initiation and duration of folic acid, respectively

Controlling for region, maternal age, ethnicity, parity, conception mode, education level, family income, history of adverse pregnancy outcomes, medicine exposure, and early pregnancy conditions.
